# Acquisition and Processing of Brain Signals

**DOI:** 10.3390/s21196492

**Published:** 2021-09-29

**Authors:** Andrea Bizzego, Gianluca Esposito

**Affiliations:** 1 Department of Psychology and Cognitive Science, University of Trento, 38068 Trento, Italy; 2 Psychology Program, Nanyang Technological University, Singapore 639818, Singapore; 3 Lee Kong Chian School of Medicine, Nanyang Technological University, Singapore 308232, Singapore

We live within a context of unprecedented opportunities for brain research, with a flourishing of novel sensing technologies and methodological approaches. Technological progress has made available a new generation of sensors and devices that facilitate the collection of central and peripheral nervous system signals [1,2,3]. Physiological signals are most efficiently analyzed by means of modern analysis techniques, particularly those based on data fusion and deep learning algorithms [4].

For this Special Issue (SI), we have collected articles that focus on comparisons of the sensing technologies and on standardization of the signal processing procedures to collect and analyze central and peripheral nervous system signals. These studies describe the use of new technologies and technological advancements, both in terms of signal processing methods and innovative applications. The articles of this SI can be grouped into three categories: tutorials and reviews, including three papers that present the current status of the literature in the analysis of cardiac and functional near-infrared spectroscopy (fNIRS) signals; signal processing, including four papers that introduce novel methods and approaches; and sensors and applications, including four papers that highlight potential applications of wearable devices in different clinical and research domains.

Within the fast-paced landscape of brain signal acquisition and processing applications, contributions aimed at improving the standardization of the research are very important. This SI includes three contributions, two focusing on the analysis of cardiac signals, and another targeting hyperscanning experiments using fNIRS signals.

Notwithstanding the importance of heart rate variability (HRV) analysis in research, users with no adequate background might find it difficult to identify and implement the analytical steps required to obtain HRV measures. To address this issue, Pham and colleagues [5] provided a review of the state-of-the-art methods for HRV analysis, and a tutorial to implement them using NeuroKit2 [6], a Python package for Neurophysiological Signal Processing.

HRV measures are also used to identify psychological states, for instance, cognitive fatigue. On this topic, Lee and colleagues [7] reviewed the current machine learning (ML)-based approaches to assess fatigued states and reflected on future directions to be explored.

Recently, research in neuroscience has started to consider the effects of inter-personal interactions on the autonomic and central brain activity [8,9]. Working with data from dyads, instead of single subjects, poses some challenges; furthermore, experiments with fNIRS require standardization [10,11], but presently leave several implementation decisions to the researcher’s discretion. Nguyen and colleagues [12] offer valuable guidelines for implementing reproducible pipelines for the processing and analysis of fNIRS signals in parent–child experimental settings.

Four contributions in this SI proposed new methods for the analysis of brain signals and data from neuroscience studies. Noticeably, all of them are based on artificial intelligence (AI) approaches, which are clearly the new frontier for brain signal processing. Pantazis and Amir [13] used deep learning (DL) for the localization of magnetoencephalography (MEG) brain signals, which improved the results in terms of predictive performance, robustness and computation time compared to the standard RAP-MUSIC localization algorithm [14]. Gabrieli and colleagues [15] proposed a data quality control pipeline based on ML, to discriminate between usable and unusable eye fixation recordings. The results prove that the proposed approach could be effectively used to label collected data. Prasanna and colleagues [16] used DL to discriminate between focal and non-focal electroencephalogram (EEG) signals, which is crucial to localizing the epileptogenic zone during neurosurgery. The network processes entropy features and frequency features computed with the fast Walsh–Hadamard transform [17], and demonstrated state-of-the-art performance on two reference datasets. Lai and colleagues [18] focused on the discrimination of patients with non-severe traumatic brain injury and healthy subjects based on EEG signals. They proposed the use of a hybrid model, composed of a DL network, including a long-short term memory layer, which is used to automatically extract features that are provided to an error-correcting output coding support vector machine.

Finally, this SI includes four contributions focusing on potential applications of new sensors, particularly wearable devices. Sela and colleagues [19] reflected on the technological and ethical challenges of a novel multi-domain socio-ecological model of health, based on the integration of data from neuro-sensors and psychosocial metrics. Kennedy-Metz and colleagues [20] offered a proof of concept of the integrated use of heart rate and fNIRS sensing in a critical setting, a cardiac surgery operating room. Specifically, this use case focused on identifying the influence of intra-operative events on cardiovascular and prefrontal cortex changes. Bizzego and colleagues [21] presented a framework for the validation of devices for physiological signal acquisition for research purposes. In particular, the study dealt with the issue of assessing the replicability of physiological measures, when computed on lower quality data from wearable devices. Al-Ezzi and colleagues [22] analyzed the associations among brain connectivity, measured with EEG sensors, and the severity of social anxiety disorders. The outcomes of this study shed new light on the comprehension of neural biomarkers underlying social anxiety disorders, paving the way to future clinical applications.

The contributions of this SI regarding brain signal acquisition and processing reveal a vital and fruitful research field, which, besides the standard approaches and paradigms, is open to novel models and methods.

This particular context is the result of the co-occurrence of two favorable circumstances: (a) the availability of affordable and portable sensors and devices, and (b) the flourishing of new processing methods based on AI. New sensors and devices reduce the physical constraints on the acquisition of brain signals and allow focusing on novel research contexts. AI methods facilitate the analysis of collected data, providing more powerful and effective methods. Together, they are re-shaping the field of brain signal acquisition and processing.

More than ever, within this context of new opportunities, reproducibility is a key aspect that should be the priority of all researchers working in this domain. To maximize the impact of the research, the advancement of the sensing and processing technologies should be associated with the adoption of rigorous and standardized methods which favor the comparability and reproducibility of the findings.
